# Family Burden, Emotional Distress and Service Satisfaction in First Episode Psychosis. Data from the GET UP Trial

**DOI:** 10.3389/fpsyg.2017.00721

**Published:** 2017-05-16

**Authors:** Mirella Ruggeri, Antonio Lasalvia, Paolo Santonastaso, Francesca Pileggi, Emanuela Leuci, Maurizio Miceli, Silvio Scarone, Stefano Torresani, Sarah Tosato, Katia De Santi, Doriana Cristofalo, Carla Comacchio, Simona Tomassi, Carla Cremonese, Angelo Fioritti, Giovanni Patelli, Chiara Bonetto, The GET UP Group

**Affiliations:** ^1^UOC Psichiatria, Azienda Ospedaliera Universitaria IntegrataVerona, Italy; ^2^Department of Neurosciences, Biomedicine and Movement Sciences, Section of Psychiatry, University of VeronaVerona, Italy; ^3^Department of Neurosciences, University of Padova and Azienda OspedalieraPadova, Italy; ^4^Department of Mental Health, Azienda USL BolognaBologna, Italy; ^5^Department of Mental Health, Azienda USL ParmaParma, Italy; ^6^Department of Mental Health, Azienda USL FirenzeFirenze, Italy; ^7^Department of Psychiatry, University of MilanoMilan, Italy; ^8^Department of Mental HealthAzienda USL Bolzano, Italy; ^9^AO Ospedale Niguarda Ca' Granda Milano, MHD Programma2000Milan, Italy

**Keywords:** early psychosis, relatives, outcomes, pragmatic trial, community psychiatry

## Abstract

**Background:** Literature has documented the role of family in the outcome of chronic schizophrenia. In the light of this, family interventions (FIs) are becoming an integral component of treatment for psychosis. The First Episode of Psychosis (FEP) is the period when most of the changes in family atmosphere are observed; unfortunately, few studies on the relatives are available.

**Objective:** To explore burden of care and emotional distress at baseline and at 9-month follow-up and the levels of service satisfaction at follow-up in the two groups of relatives (experimental treatment EXP vs. treatment as usual TAU) recruited in the cluster-randomized controlled GET UP PIANO trial.

**Methods:** The experimental treatment was provided by routine public Community Mental Health Centers (Italian National Health Service) and consisted of Treatment as Usual plus evidence-based additional treatment (Cognitive Behavioral Therapy for psychosis for patients, Family Intervention for psychosis, and Case Management). TAU consisted of personalized outpatient psychopharmacological treatment, combined with non-specific supportive clinical management and informal support/educational sessions for families. The outcomes on relatives were assessed by the Involvement Evaluation Questionnaire (IEQ-EU), the General Health Questionnaire (GHQ-12), and the Verona Service Satisfaction Scale (VSSS-EU). Differences within and between groups were evaluated.

**Results:** At baseline, 75 TAU and 185 EXP caregivers were assessed. In the experimental group 92% of relatives participated in at least 1 family session. At follow-up both groups experienced improvement in all IEQ and GHQ items, but caregivers belonging to the EXP arm experienced a significantly greater change in 10 IEQ items (mainly pertaining to the “Tension” dimension) and in GHQ items. Due to the low sample size, a significant effectiveness was only observed for 2 IEQ items and 1 GHQ-12 item. With respect to VSSS data at follow-up, caregivers in the EXP arm experienced significantly greater satisfaction in 8 items, almost all pertaining to the dimensions “Relatives' Involvement” and “Professionals' Skills and Behavior.”

**Conclusions:** The Family intervention for psychosis delivered in the GET UP PIANO trial reduced family burden of illness and improved emotional distress and satisfaction with services. These results should encourage to promote FIs on caregivers of first-episode psychosis patients.

## Introduction

International literature has explored the role of family in the outcome of chronic schizophrenia, but only few studies are available on the relatives of people experiencing their first episode of psychosis (FEP). This is a period when most of the changes in family dynamics are observed (Addington et al., 2003; Koutra et al., 2014; Yesufu-Udechuku et al., 2015).

Schizophrenia impacts not only on the patients but also to those who care for them. The term caregiving burden since the 80s has been used to explore these consequences (Hatfield and Lefley, 1987) and contributed to highlight. Family members of persons with a severe mental disorder experience negative effects linked to these burdensome situations (Budd et al., 1998; Magliano et al., 2005).

With the shift from hospital-based to community-based care which occurred in most western countries, the relatives of mentally ill people have become an integral part of the care system. Thus, taking into account the carers' perspectives on mental health provision and on overall families' needs and support has become of crucial importance.

Research has shown that about one third of the relatives of schizophrenic patients suffer from emotional or behavioral distress and that patterns of familiar interpersonal interaction can worsen the course of schizophrenia (Magliano et al., 2000; Moller-Leimkuhler, 2006). Several studies have explored carers' needs and perceptions, coping styles, mental health and satisfaction. Follow-up studies on the burden on relatives of persons affected by psychosis treated in community-based routine mental health services are uncommon. Even less studies on the burden that develops in the early stages of psychosis, and on the impact of the interventions provided (Brown and Birtwistle, 1998; Magliano et al., 2000; Moller-Leimkuhler, 2006; Breitborde et al., 2011) have been conducted. These follow-up studies have not provided clear information on the patterns in family burden and thus, there is lack of consensus on burden in the medium long term and also on which specific factors could impact on it, and at which stages.

The GET UP (Genetics, Endophenotypes, Treatment: Understanding early Psychosis) PIANO (Psychosis: early Intervention and Assessment of Needs and Outcome) Trial (Ruggeri et al., 2012, 2015) was conducted in a large epidemiologically based cohort from Italian community mental health centers (CMHCs). It aimed to assess the feasibility and effectiveness of a multi-element psychosocial intervention for FEP patients and their families, compared with treatment as usual (TAU). Experimental treatment was administered as an adjunct to TAU, and included: (1) cognitive behavioral therapy for psychosis (CBTp) to patients; (2) psychosis-focused family intervention (FIp) to families; and (3) case management (CM) to both patients and relatives. The intervention was provided by CMHC staff, trained in the previous 6 months and supervised by experts.

It was hypothesized that in the patients add-on multi-component intervention could favor: (1) greater improvements in symptoms, as measured by the PANSS; and (2) reduction in days of hospital admission over the 9-month follow-up. Secondary hypotheses were that the intervention could improve subjective burden of psychotic symptoms (auditory hallucinations and delusions), social functioning and emotional well-being and produce lower service disengagement rates (Ruggeri et al., 2015).

The hypotheses regarding the relatives were that the multi-element experimental intervention at the 9 month follow-up could: (1) reduce burden of the key relative, as measured using the IEQ; and (2) improve service satisfaction, as measured using the VSSS Relatives scale (Ruggeri and Dall'Agnola, 1993), at follow-up.

In the present paper we aim to provide comprehensive descriptives on the effect on relatives of the 9 month multi-element psychosocial intervention for FEP patients and their families in routine MH services versus treatment as usual (TAU). Specifically we address: (a) changes in the relatives' burden of care and emotional distress and (b) differences in the levels of service satisfaction reached at the end of the follow-up. Testing these hypotheses seems to be of particular relevance in a cultural context, such as Italy, where the majority of the patients continue to live with their families even for long time after the mental illness onset, with potential severe consequences on the everyday life of those caring families.

## Methods

### Study design

The assignment units (clusters) in the GET UP cluster were the CMHCs and the units of observation and analysis were patients and their families (Ruggeri et al., 2012, 2015). Participation was offered to all CMHCs serving two northern Italian regions (Veneto and Emilia-Romagna) and the urban areas of Florence, Milan, and Bolzano, covering an area of 9,951,306 inhabitants. Of 126 CMHCs, 117 (92.8%, covering 9,304,093 inhabitants) participated.

### Service organization context and participating sites

MH care in Italy is organized in catchment areas, where one or more CMHCs provide outpatient care, daycare, and rehabilitation to nearly 100,000 inhabitants. It is delivered by the National Health Service through the Departments of Mental Health (DMH).

### Randomization

Stratified randomization of CMHCs took into account catchment area size, and its geographical characteristics (urban/mixed versus rural context) and was performed to balance differences in their characteristics. Also organizational constraints (such as affiliation to the same DMH) were taken into account. One CMHC each in the intervention and TAU arms withdrew consent to participate and were excluded.

### Participants

All CMHCs were asked to refer potential psychosis cases at first contact during the index period to the study team. Based on the WHO 10-country study (Jablensky et al., 1992), the inclusion criteria to ascertain FEP patients were:

- age 18–54 years
- residence in catchment areas of CMHCs
- presence of at least 1 of the following: hallucinations, delusions, qualitative speech disorder, qualitative psychomotor disorder, bizarre, or grossly inappropriate behavior, or 2 of the following: loss of interest, initiative, and drive; social withdrawal; episodic severe excitement; purposeless destructiveness; overwhelming fear; or marked self-neglect, per the WHO Screening Schedule for Psychosis (World Health Organization, 1992)
- first lifetime contact with CMHCs, prompted by these symptoms

Exclusion criteria were: (a) anti-psychotic medication (>3 months) prescribed for an identical or similar mental disorder; (b) mental disorders due to general medical condition; (c) moderate-severe mental retardation per a clinical functional assessment; and (d) psychiatric diagnosis other than ICD-10 for psychosis.

All eligible patients, identified as those who reached the clinical stabilization, were invited to provide written informed consent to be assessed and informed of the nature, scope, and possible consequences of the trial and that they could withdraw consent at any time. Patients were asked to give consent for family member assessments; family members who agreed to participate provided written informed consent.

The trial received approval by the ethics committees of the coordinating center (Azienda Ospedaliera Universitaria Integrata di Verona) and each participating unit and was registered with ClinicalTrials.gov (NCT01436331).

### Diagnostic ascertainment of patients

Since FEP is generally a phase of high diagnostic instability, the specific ICD-10 codes for psychosis (F1x.4; F1x.5; F1x.7; F20–29; F30.2, F31.2, F31.5, F31.6, F32.3, F33.3) were assigned at 9 months. The best-estimate ICD-10 diagnosis was made by consensus of a panel of clinicians by considering all available information on the time interval from patient's intake needed to apply the Item Group Checklist (IGC) of the Schedule for Clinical Assessment in Neuropsychiatry (SCAN, World Health Organization, 1992).

### Treatments

#### Experimental treatment (TAU+CBT+FI+CM)

The experimental treatment package was provided by routine public Community Mental Health Centers (CMHCs) which operate within the Italian National Health Service and consisted of standard care (treatment as usual, TAU) plus evidence-based additional treatment. Specifically, the multi-element psychosocial intervention, adjunctive to TAU, comprised: (i) Cognitive Behavioral Treatment for psychosis (CBTp) to patients; (ii) psychosis-focused Family Intervention (FIp) to individual families; and (iii) Case Management (CM) to both parties. FIp was based on the model proposed by Leff et al. (1985) and further developed by Kuipers et al. (2002). It included an optimal number of 10–15 sessions over 9 months, with each individual family: 6 sessions in the first 3 months, and at least 1 session/month in the 6 months afterwards. Every patient/family had a dedicated CM, who coordinated all planned interventions (Burns and Firn, 2002).

#### Treatment as usual (TAU)

Treatment as usual (TAU) was also provided by routine public Community Mental Health Centers (CMHCs) involved in the Trial. In Italy standard care for FEP patients typically consisted of personalized outpatient psychopharmacological treatment, combined with non-specific supportive clinical management and non-specific informal support/educational sessions for families.

### Outcome measures

The outcomes on relatives were assessed by the Involvement Evaluation Questionnaire, European Version (IEQ-EU), the 12-item General Health Questionnaire (GHQ-12), and the Verona Service Satisfaction Scale, European Version (VSSS-EU).

Specifically, the IEQ-EU is an 81-item self-rated questionnaire completed by the caregiver (van Wijngaarden et al., 2000). The questionnaire consists of seven distinct sections which assess several burden related aspects in the foregoing 4 weeks. All items are scored on a 3 or 5 point Likert scale, and can be summarized into four distinct scales: (I) “tension” (9 items)—strained interpersonal atmosphere between patient and caregiver, such as quarrels; (II) “supervision” (5 items)—caregiver's tasks of guarding the patient, for instance to prevent suicide or to supervise the intake of medicine; (III) “worrying” (6 items)—caregiver's concern about the patient's safety, future, and health; (IV) “urging” (8 items), the need to stimulate the patient to undertake activities. The other six sections of the IEQ include items which assess background information on patient, caregiver and mutual relationship. These sections are: (a) general information on patient, caregiver and household composition, time spent together (15 items); (b) costs (8 items); (c) the General Health Questionnaire to assess caregiver's distress, 12 items version (GHQ-12) (Goldberg and Williams, 1988); (d) caregiver's use of professional help (3 items); (e) consequences for patient's children (11 items); and (f) an open question for comments and additions.

Satisfaction with mental health services was assessed using the Verona Service Satisfaction Scale (VSSSR; Ruggeri and Dall'Agnola, 1993; Ruggeri et al., 1994, 1996, 2007) which is designed for self-administration and can be completed without prior training in 20–30 min. The VSSS has been implemented both in the version for Patients and for Relatives (Ruggeri et al., 2007). The VSSS Relatives consists of 54 items, which conceptually cover seven dimensions: “Overall satisfaction,” “Professionals” skills, and “behavior,” “Information,” “Access,” “Efficacy,” “Types of intervention,” and “Relative's involvement.” Relatives were asked to give overall rating about their experience of the mental health services they have attended. Satisfaction ratings are on a 5-point Likert scale (1 = terrible, 2 = mostly unsatisfactory, 3 = mixed, 4 = mostly satisfactory, 5 = excellent). The items are presented with alternate directionality to reduce stereotypic responses.

### Statistical analyses

Descriptives were calculated [mean (sd) for continuous and n (%) for categorical variables, respectively]. Comparisons between characteristics and outcome of relatives assigned to the two groups (TAU and EXP) were performed by using Fisher's exact tests for categorical items and independent samples *t*-tests for continuous scores. Changes between baseline and follow-up within each group were evaluated by using repeated measures *t*-tests and the difference between groups was explored by independent *t*-tests. All tests were bilateral at *p* < 0.05. No correction for multiple comparisons was performed due to the explorative nature of the study. All analyses were executed by SPSS for Windows (version 22).

## Results

**Figure 1:**
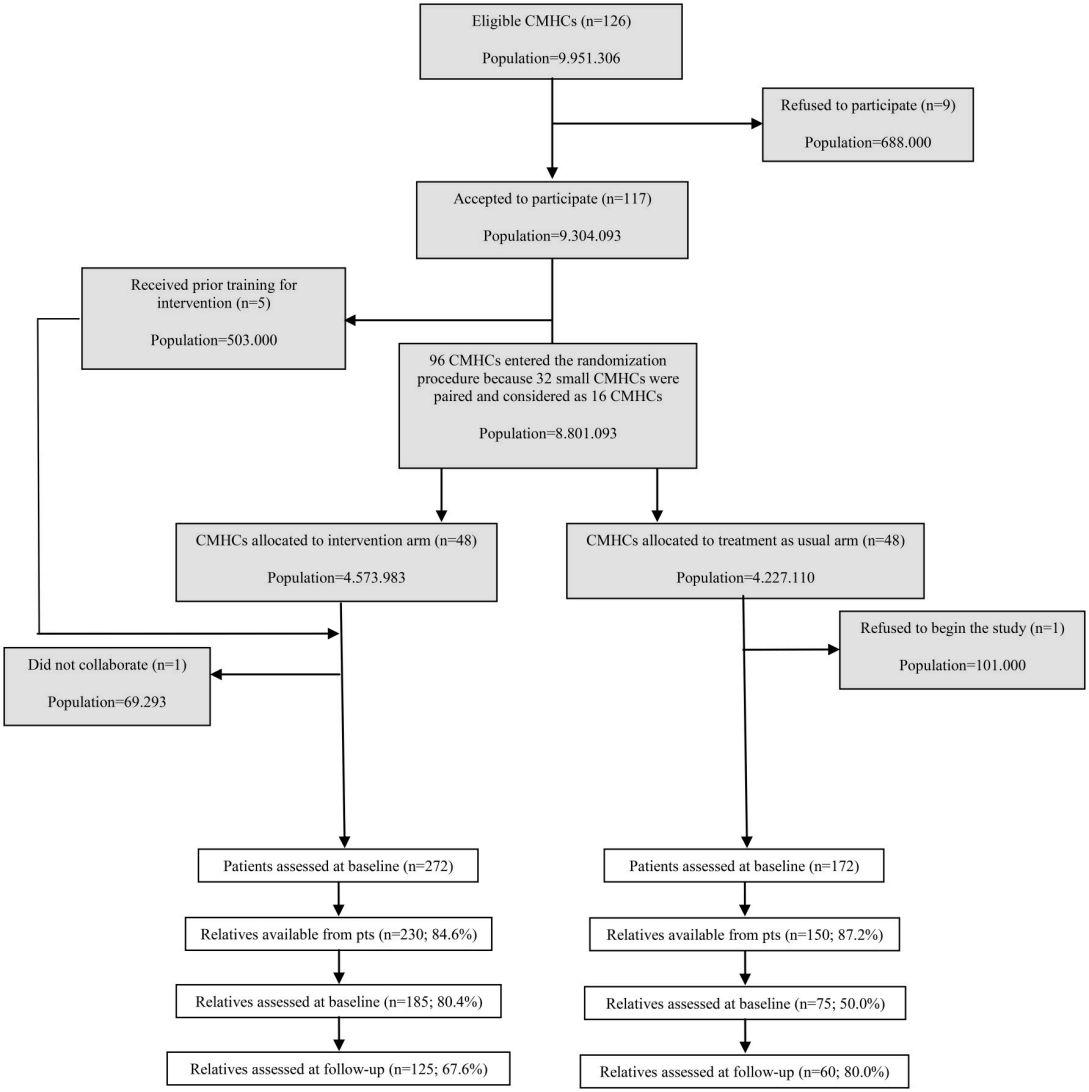
**Trial profile for relatives**.

In the experimental arm out of the 272 patients enrolled, 16 did not have a relative; in 6 cases patients did not give the permission to contact the relative; in 7 cases the relatives did not want to undergo into Family Intervention and in 13 cases the patient refused to enter in the CBT intervention, and thus the relative was also excluded. In the TAU arm, out of the 172 patients enrolled, 10 did not have a relative; in 12 cases patients did not give the permission to contact the relative. Thus, it has been possible to ask the permission to be assessed to 230 relatives in the experimental arm and to 150 relatives in the TAU arm. Of these, 185 subjects in the experimental arm (out of 230 available; 80%) and 75 in the TAU arm (out of 150 available; 50%) accepted to complete the trial's baseline assessments (Figure 1).

Relatives in the two groups did not differ in any socio-demographic characteristics. No other between-group differences were observed at baseline in any variable concerning cohabitation with the patient (see Table 1). It is noteworthy that the majority of the relatives were parents of the affected patient, and that the largest majority lived with the patient and were used to stay with the patient all days and for several hours.

**Table 1:** **Socio-demographics of relatives assessed at baseline and cohabitation with patient variables**.

	**Baseline**		
	**Treatment as usual group (*n* = 75)**	**Experimental treatment group (*n* = 185)**	**Test and significance of difference**
**Gender**, ***n*** **(%)**			
Male	28 (37.3%)	69 (37.3%)	χ^2^ = 0.00, *df* = 1, *p* = 0.980
Female	47 (62.7%)	115 (62.7%)	
**Age, mean (sd)**	50.7 (10.5)	49.6 (11.2)	*t* = 0.73, *df* = 258, *p* = 0.466
**Education**, ***n*** **(%)**	(1 missing)		
Low level	40 (54.1%)	81 (43.8%)	χ^2^ = 2.24, *df* = 1, *p* = 0.135
High level	34 (45.9%)	104 (56.2%)	
**Marital status**, ***n*** **(%)**			
Unmarried	7 (9.3%)	12 (6.5%)	χ^2^ = 2.65, *df* = 2, *p* = 0.265
Married	53 (70.7%)	148 (80.0%)	
Widowed, separated, divorced	15 (20.0%)	25 (13.5%)	
**Living status**, ***n*** **(%)**	(2 missing)	(5 missing)	
Alone	1 (1.4%)	4 (2.2%)	χ^2^ = 1.04, *df* = 4, *p* = 0.904
With partner and/or children With parents and/or siblings With other relatives	63 (86.3%)3 (4.1%)3 (4.1%)	157 (87.2%)6 (3.3%)4 (2.2%)	
Other	3 (4.1%)	9 (5.0%)	
**Relationship with patient**, ***n*** **(%)**		(2 missing)	
Mother/Father	47 (62.7%)	121 (66.1%)	χ^2^ = 1.52, *df* = 4, *p* = 0.821
Daughter/Son	3 (4.0%)	6 (3.3%)	
Sister/Brother	9 (12.0%)	14 (7.6%)	
Wife/Husband Friend/Other	15 (20.0%)1 (1.3%)	38 (20.8%)4 (2.2%)	
**Patient's gender**, ***n*** **(%)**		(1 missing	
Male	47 (62.7%)	113 (61.4%)	χ^2^ = 0.04, *df* = 1, *p* = 0.851
Female	28 (37.3%)	71 (38.6%)	
**Patient's age, mean (sd)**	30.2 (8.9)	(2 missing) 28.2 (9.9)	*t* = 1.52, *df* = 256, *p* = 0.131
**Years from the beginning of mental disorder**, ***n*** **(%)** ≤ 1 1–5 5–10 >10	(4 missing) 35 (49.3%) 26 (36.6%)3 (4.2%)7 (9.9%)	(12 missing) 97 (56.1%)60 (34.7%)8 (4.6%)8 (4.6%)	χ^2^ = 2.74, *df* = 3, *p* = 0.433
**Is patient receiving help for mental disorder now?** ***n*** **(%)**		
Yes	73 (94.7%)	183 (98.9%)	χ^2^ = 0.89, *df* = 1, *p* = 0.347
No	2 (5.3%)	2 (1.1%)	
**Relative lives with patient**, ***n*** **(%)**		(1 missing)	
No	9 (12.0%%)	14 (7.6%)	χ^2^ = 1.24, *df* = 1, p = 0.266
Yes	66 (88.0%)	169 (92.4%)	
**Days per week in the last 4 weeks**, ***n*** **(%)**		(2 missing)	
Never	9 (12.0%)	12 (6.6%)	χ^2^ = 4.16, *df* = 2, p = 0.125
Some days	3 (4.0%)	18 (9.8%)
All days	63 (84.0%)	153 (83.6%)	
**Hours per week in the last 4 weeks**, ***n*** **(%)**		(5 missing)	
0–4 5–8 9–16 17–32 >32	2 (2.7%)7 (9.3%)7 (9.3%)8 (10.7%)51 (68.0%)	7 (3.9%)10 (5.6%)21 (11.7%)16 (8.8%)126 (70.0%)	χ^2^ = 1.83, *df* = 4, *p* = 0.766

Engagement of the relatives in the Family intervention was good: as shown in Table 2, in the experimental group 170 out of 185 relatives assessed at BL (91.9%) participated in at least 1 family session. Most of them (*n* = 154; 90.6%) received ≥5 FI sessions; of these 112 (72.7%) received ≥10 sessions.

**Table 2:** **Intervention provision (Family intervention and non-specific interventions) in the relatives assessed at BL**.

	**Period between BL and FU**	
	**Treatment as usual group (n = 75)**	**Experimental treatment group (n = 185)**
**Family intervention sessions**, ***n*** **(%)** 0 1–4 5–9 10–19 20+	————–	15 (8.1%) 16 (8.6%) 42 (22.7%) 94 (50.8%) 18 (9.8%)
**Families receiving non-specific interventions**, ***n*** **(%)**	(5 missing) 21 (30.0%)	(12 missing) 15 (8.7%)
**Types of non-specific interventions**, ***n*** **[mean (sd)]**	(3 missing)	(3 missing)
Psychological support	8 [4.2 (2.9)]	11 [3.5 (2.4)]
Non-specific psychoeducation	4 [9.7 (5.9)]	–
Systemic psychotherapy	2 [6.0 (4.2)]	1 [5.0 (.)]
Other	4 [6.5 (6.4)]	–

At follow-up 75 relatives could not be traced and thus could not be assessed: 60 subjects belonged to the experimental arm (32.4% of the cohort) and 15 to the TAU arm (20.0% of the cohort). There were no significant differences in demographics and outcome variables at baseline between completers and non-completers. Both groups had similar baseline levels of burden of care. At follow-up both groups experienced a general trend to improvement in most IEQ items.

However, as shown in Table 3, caregivers belonging to the experimental arm at the 9 month follow-up had significantly greater improvement in burden caused by a series of aspects of everyday life with the patient: “Encouraged to eat,” “Ensured medicine,” “Guarded dangerous acts,” “Guarded taking drugs,” “Disturbed sleep,” “Atmosphere strained?,” “Caused quarrel,” “Annoyed Behavior,” “Others annoyed,” and “Burden” (see Table 3). The majority of these aspects pertains to the “Tension” dimension. The effectiveness of the treatment reached significance only for “Disturbed sleep” and “Atmosphere strained?.”

**Table 3:** **Mean scores (sd) of burden of care (IEQ-EU) for relatives assessed at baseline and at 9-month follow-up (dimensions: S, Supervision; T, Tension; U, Urging; W, Worrying)**.

**IEQ-EU items**	**Treatment as usual group**			**Experimental treatment group**			**Δ (FU-BL) TAU vs. EXP**
	**BL (*n* = 60)**	**FU (*n* = 60)**	***p*-value paired *t*-test**	**BL (*n* = 125)**	**FU (*n* = 125)**	***p*-value paired *t*-test**	***p*-value *t*-test**
Encouraged proper care (U)	2.33 (1.44)	1.82 (1.18)	0.012^**^	2.09 (1.23)	1.82 (1.12)	0.029^*^	0.306
Helped proper care (U)	1.67 (1.22)	1.41 (0.88)	0.150	1.45 (1.01)	1.32 (0.84)	0.204	0.542
Encouraged to eat (U)	1.52 (0.97)	1.30 (0.46)	0.102	1.63 (1.07)	1.33 (0.78)	0.006^**^	0.696
Encouraged activity (U)	2.82 (1.32)	2.35 (1.25)	0.002^**^	2.53 (1.14)	2.24 (1.18)	0.021^*^	0.380
Accompanied activity (U)	1.76 (1.20)	1.32 (0.84)	0.014^**^	1.74 (1.11)	1.27 (0.65)	0.000^**^	0.856
Ensured medicine (U)	2.78 (1.74)	2.46 (1.76)	0.182	2.90 (1.79)	2.05 (1.64)	0.000^**^	0.121
Guarded dangerous acts (S)	1.51 (1.17)	1.27 (0.90)	0.219	1.56 (1.15)	1.24 (0.82)	0.002^**^	0.646
Guarded self-harm (S)	1.61 (1.18)	1.29 (0.92)	0.038^*^	1.66 (1.20)	1.17 (0.71)	0.000^**^	0.370
Ensured sleep (S)	2.12 (1.38)	1.73 (1.27)	0.007^**^	2.35 (1.46)	1.64 (1.00)	0.000^**^	0.182
Guarded alcohol (S)	1.78 (1.35)	1.43 (1.10)	0.048^*^	1.60 (1.25)	1.48 (1.03)	0.304	0.272
Guarded taking drugs (S)	1.49 (1.22)	1.29 (0.90)	0.222	1.58 (1.29)	1.36 (0.98)	0.034^*^	0.890
Carried out tasks (U)	1.78 (1.08)	1.49 (0.89)	0.068	1.77 (1.09)	1.53 (0.96)	0.067	0.829
Get up in the morning (U)	2.08 (1.26)	2.08 (1.31)	1.000	2.04 (1.26)	2.22 (1.40)	0.181	0.483
Disturbed sleep (T, S)	1.25 (0.56)	1.33 (0.95)	0.591	1.48 (0.96)	1.20 (0.59)	0.004^**^	0.041^a^
Atmosphere strained (T)	1.75 (0.84)	1.76 (0.91)	0.868	1.90 (0.99)	1.59 (0.69)	0.001^**^	0.045^a^
Caused quarrel (T)	1.43 (0.79)	1.51 (0.94)	0.498	1.54 (0.89)	1.36 (0.66)	0.032^*^	0.084
Behavior annoyed (T)	1.80 (0.88)	1.74 (0.94)	0.679	1.74 (0.96)	1.55 (0.77)	0.041^*^	0.446
Others annoyed (T)	1.20 (0.45)	1.16 (0.42)	0.598	1.33 (0.71)	1.12 (0.47)	0.000^**^	0.078
You felt threatened (T)	1.08 (0.28)	1.02 (0.14)	0.083	1.13 (0.46)	1.05 (0.26)	0.083	0.833
Moving out behavior (T)	1.16 (0.51)	1.16 (0.47)	1.000	1.26 (0.76)	1.21 (0.76)	0.433	0.636
Pursue own activities	2.92 (1.44)	3.06 (1.53)	0.566	3.05 (1.46)	3.32 (1.53)	0.169	0.714
Worried about safety (W)	2.32 (1.50)	1.70 (0.97)	0.001^**^	2.59 (1.45)	1.85 (1.31)	0.000^**^	0.643
Worried treatment received (W)	2.56 (1.40)	2.04 (1.29)	0.016^*^	2.58 (1.34)	1.87 (1.19)	0.000^**^	0.483
Worried general health (W)	3.37 (1.36)	2.57 (1.39)	0.000^**^	3.50 (1.25)	2.52 (1.44)	0.000^**^	0.491
Worried manage financially (W)	2.80 (1.55)	2.22 (1.28)	0.010^**^	2.25 (1.44)	2.05 (1.30)	0.142	0.124
Worried relative/friend's future (W)	3.76 (1.21)	3.02 (1.35)	0.000^**^	3.31 (1.34)	2.64 (1.35)	0.000^**^	0.760
Worried own future (T)	2.82 (1.60)	2.59 (1.43)	0.274	2.51 (1.39)	2.24 (1.30)	0.068	0.898
Burden (T, W)	1.75 (0.91)	1.58 (0.99)	0.197	2.02 (1.06)	1.64 (1.02)	0.000^^**^^	0.236

* *p < 0.05*,

** *p < 0.01*.

a *Cohen's d = 0.34*.

Emotional distress at baseline, as measured by GHQ, was similar in both groups. As shown in Table 4, at follow-up both groups experienced a general trend for improvement in emotional distress. However, caregivers belonging to the experimental arm had a significantly greater improvement in the majority of GHQ items, with the only exception of “Feeling reasonably happy” (Table 4) where improvement was equally significant in the two groups. The effectiveness of the treatment reached significance only for “Under strain.”

**Table 4:** **Mean scores (sd) of emotional distress (GHQ-12) for relatives assessed at baseline and at 9-month follow-up**.

**GHQ-12 items**	**Treatment as usual group**			**Experimental treatment group**			**Δ (FU-BL) TAU vs. EXP**
	**BL (*n* = 60)**	**FU (*n* = 60)**	***p*-value paired *t*-test**	**BL (*n* = 125)**	**FU (*n* = 125)**	***p*-value paired *t*-test**	***p*-value *t*-test**
Concentrate on what doing	2.26 (0.72)	2.18 (0.52)	0.420	2.33 (0.76)	2.10 (0.48)	0.004^**^	0.264
Lost much sleep	2.41 (1.02)	2.12 (0.97)	0.050	2.54 (1.02)	1.90 (0.87)	0.000^**^	0.056
Useful part in things	1.57 (0.57)	1.88 (0.65)	0.004^**^	1.86 (0.77)	1.95 (0.55)	0.304	0.129
Making decisions	1.84 (0.54)	1.94 (0.54)	0.280	1.96 (0.62)	1.99 (0.43)	0.608	0.602
Under strain	2.61 (1.00)	2.41 (1.00)	0.176	2.88 (0.96)	2.19 (0.87)	0.000^**^	0.007^**^^a^
Overcome difficulties	2.20 (1.06)	1.94 (0.99)	0.108	2.27 (1.02)	1.84 (0.80)	0.000^**^	0.359
Enjoy normal activities	2.29 (0.78)	2.14 (0.53)	0.146	2.42 (0.71)	2.09 (0.41)	0.000^**^	0.180
Face up to problems	2.14 (0.57)	2.12 (0.47)	0.811	2.17 (0.64)	1.99 (0.36)	0.013^**^	0.183
Unhappy or depressed	2.33 (1.07)	2.14 (1.02)	0.215	2.43 (1.06)	1.91 (0.97)	0.000^**^	0.063
Losing confidence	1.65 (0.87)	1.55 (0.81)	0.389	1.58 (0.88)	1.45 (0.80)	0.198	0.848
Worthless person	1.33 (0.65)	1.43 (0.78)	0.471	1.27 (0.63)	1.25 (0.63)	0.807	0.409
Reasonable happy	2.59 (0.83)	2.22 (0.67)	0.001^**^	2.63 (0.81)	2.12 (0.61)	0.000^**^	0.336

^*^p < 0.05,

** *p < 0.01*.

a *Cohen's d = 0.46*.

Caregivers in the experimental arm experienced significantly greater levels of service satisfaction after the 9 month treatment in “Ability of psychiatrists to listen and understand patients,” “Recommendations to the closest relative,” “Relatives' knowledge to problems,” “How information is given on diagnosis and prognosis,” “Ability of psychiatrists to listen and understand relatives,” “Thoroughness of nurses,” “Helping relatives,” and “Continuity of care” (see Table 5). All these aspects pertain to two dimensions (“Relatives' Involvement” and “Professionals' Skills and Behavior”), with the only exception of “How information is given on diagnosis and prognosis.”

**Table 5:** **Mean scores (sd) and n (%) of service satisfaction (VSSS-EU) for relatives assessed at 9-month follow-up (dimensions: A, Access; E, Efficacy; I, Information; OS, Overall Satisfaction; PSB, Professionals' Skills and Behavior; RI, Relatives' Involvement; TI, Types of Intervention)**.

**VSSS-EU items**	**Treatment as usual group**	**Experimental treatment group**	
	**FU (*n* = 60)**	**FU (*n* = 125)**	***p*-value *t*-test or Fisher's exact test**
Effectiveness of service in helping (E)	4.15 (0.74)	4.17 (0.66)	0.867
Behavior of secretary staff (PSB)	4.33 (0.82)	4.34 (0.70)	0.925
Professional competence of psychiatrists/psychologists (PSB)	4.33 (0.71)	4.36 (0.65)	0.822
Comfort facilities (A)	3.97 (0.72)	4.01 (0.78)	0.725
Ability of psychiatrists/psychologists to listen and understand patient (PSB)	4.20 (0.64)	4.41 (0.70)	0.049^*^
Manners of psychiatrists/psychologists (PSB)	4.37 (0.84)	4.46 (0.63)	0.441
Punctuality of professionals (PSB)	4.15 (0.82)	4.14 (0.82)	0.928
Cost of service (A)	4.46 (0.68)	4.45 (0.70)	0.972
Service effective in preventing relapse (E)	4.20 (0.71)	4.14 (0.79)	0.622
Respect for rights (PSB)	4.47 (0.75)	4.41 (0.68)	0.583
Help received (OS)	4.19 (0.63)	4.21 (0.72)	0.878
Explanation procedures (I)	3.92 (0.95)	4.10 (0.75)	0.163
Service effective in relieving symptoms (E)	4.11 (0.75)	4.11 (0.73)	0.985
Response of service to urgent needs (TI)	4.04 (0.77)	4.11 (0.84)	0.627
Arrangements for after hour emergencies (TI)	3.85 (0.82)	3.93 (0.94)	0.673
Thoroughness of psychiatrists/psychologists (PSB)	4.09 (0.81)	4.20 (0.74)	0.381
Referring to GP or other professionals (PSB)	4.08 (0.89)	4.04 (0.77)	0.775
Cooperation between service providers (PSB)	4.20 (0.80)	4.20 (0.76)	0.965
Publicity or information on services (I)	3.70 (1.05)	3.85 (0.80)	0.293
Kinds of service offered (OS)	4.02 (0.78)	4.11 (0.74)	0.434
Service received (OS)	4.23 (0.73)	4.24 (0.76)	0.949
Professional competence of nurses (PSB)	4.18 (0.74)	4.32 (0.64)	0.210
Recommendations to closest relative (RI)	3.88 (1.08)	4.23 (0.76)	0.013^**^
Effectiveness of service for your knowledge of problems (E)	3.93 (0.90)	4.06 (0.83)	0.352
Manners of nurses (PSB)	4.25 (0.84)	4.37 (0.69)	0.342
Effectiveness of service for improving relationship with relative (E)	3.88 (0.99)	4.04 (0.80)	0.271
Effectiveness of service for relatives' knowledge of problems (RI)	3.82 (1.05)	4.14 (0.74)	0.023^*^
Nurses knowledge about you (PSB)	3.84 (0.84)	3.89 (0.78)	0.721
How information is given on diagnosis and prognosis (I)	3.67 (1.02)	3.95 (0.76)	0.048^*^
Ability of psychiatrists/psychologists to listen and understand relative (RI)	3.86 (1.02)	4.25 (0.94)	0.012^**^
Effectiveness of service for relationships out the family (E)	3.80 (0.96)	3.99 (0.83)	0.171
How information is given to relative (RI)	3.64 (1.04)	3.82 (0.89)	0.237
Instructions on what to do between appointments (PSB)	3.93 (0.98)	4.00 (0.70)	0.589
Effectiveness of service for your self-care (E)	3.80 (1.03)	3.84 (0.86)	0.808
Thoroughness of nurses (PSB)	3.96 (0.91)	4.25 (0.60)	0.017^*^
Effectiveness of service for helping relative (RI)	3.73 (1.15)	4.05 (0.87)	0.043^*^
Ability of nurses and social workers to listen and understand patient (PSB)	3.94 (0.93)	4.19 (0.72)	0.063
Effectiveness of service for your work (E)	3.71 (1.03)	3.86 (0.83)	0.309
Help on side effects from medications (TI)	3.81 (0.91)	3.84 (0.88)	0.878
Continuity of care (PSB)	4.05 (0.89)	4.38 (0.65)	0.007^**^

* *p < 0.05*,

** *p < 0.01*.

## Discussion

Relatives of persons affected by psychosis play a crucial role in their pathways to care, being the first to request for help in the majority of the cases (Del Vecchio et al., 2015; Jansen et al., 2015). Several studies have also shown that relatives often feel despair, fears, concerns regarding professional support (Tennakoon et al., 2000; Faridi et al., 2009; Boydell et al., 2014; Lavis et al., 2015; Hickman et al., 2016; Koutra et al., 2014). This paper contributes to shed some light on the potentiality that interventions specific for the FEP have to reduce burden of care in an Italian context that is characterized for the majority of psychotic patients by a close link with they families, who continue to take care of them also for long time after the onset.

Data provided by the GET UP Trial allow to draw interesting conclusions on the role of relatives in the care of their loved ones and on the potentiality that specific forms of individual Family interventions can have already in the early phases of psychosis. The multi-element experimental intervention tested in the GET UP Trial, consisting of CBTp for the patients, Family intervention and Case management is effective in decreasing relatives' burden in several specific aspects of burden mainly related to the “Tension” dimension of the IEQ. The study has also shown that emotional distress is present in the relatives already in the first months after psychosis onset, and proved that the experimental multi-element intervention tested is effective in decreasing that emotional distress. Due to the multi-element treatment design of the study, it is not possible to disentangle the effectiveness that might be attributed to the family intervention. However, in the GET UP Trial, acceptability and engagement in family intervention was very good included a large number of families: in the experimental group 92% of relatives participated in at least 1 family session with the majority that could attend up to 10 sessions (defined as optimal standard for the 9 month treatment).

Caregivers in the experimental arm showed significantly greater satisfaction in 8 items, all pertaining to the dimensions “Relatives' Involvement” and “Professionals' Skills and Behavior,” with the only exception of “How information is given on diagnosis and prognosis,” related to the domain “Information given by the professionals.” This finding is of interest also in the light of literature findings showing that relatives often feel that their role and contribution is undervalued by the clinicians (Iyer et al., 2011; McCann et al., 2012).

Overall, the Family Intervention for psychosis delivered in the GET UP PIANO trial reduced family burden of illness and improved emotional distress and satisfaction with services. It should however be said that this improvement is uneven and that not all aspects of everyday interaction with the patient and with the service have been more positively affected in the experimental group.

Results presented in this paper have some methodological limitations. The relatively high number of relatives that did not give consent to the baseline assessment and the relatively high proportion of relatives that were non completers at follow-up is a limitation of these data even if the lack of significant differences in the sociodemographic characteristics between the two groups hampers this limitation. Moreover, the majority of the differences between changes within groups did not result significant due to the low sample size. Finally, financial constraints have compelled the GET UP trial researchers to plan a follow-up period of only 9 months: Thus, the short duration of the intervention did not allow to fully exploit the effects of treatment in improving burden and emotional distress in the relatives.

Among the strengths of this paper we first enlist the fact that 90% of CMHCs asked to participate accepted and completed the study, thus favoring high representativeness of the services and subjects included in the study. A further strength is that relatives belonging to experimental and TAU arm had similar baseline socio-demographic characteristics. Moreover, as most study's relatives live with the patients and stay with them for several hours/day proves that the trial results provide a realistic picture of problems and potentiality to benefit of treatments regarding those relatives which have the most relevant need of help.

In conclusion, data provided in this paper prove the higher effectiveness over TAU of a specific evidence-based multi-element treatment including family intervention in improving family burden and emotional distress. These data further strengthen previous literature on this issue (Jeppesen et al., 2005; Bird et al., 2010; Gleeson et al., 2010; Onwumere et al., 2011; Lee et al., 2014; Nordentoft et al., 2015) and encourage to promote specific forms of family interventions on caregivers of first-episode psychosis patients, and implement further studies that target their needs in the different stages of psychosis.

## Ethics statement

This study is conducted according to globally accepted standards of good clinical practice, in agreement with the Declaration of Helsinki and in keeping with local regulations.

GET UP PIANO investigators ensure that all professionals involved in the trial are adequately qualified and informed about the protocol, the study interventions, and their trial-related duties and functions. The coordinating center maintains a list of all appropriately qualified persons involved in the study.

### Ethics committee approval

Formal ethical approval for conducting the trial has been sought and obtained by the Coordinating Center's Ethics Committee (Comitato Etico per la Sperimentazione, Azienda Ospedaliera di Verona, http://www.ospedaliverona.it/Istituzionale/Comitati-Etici/Sperimentazione), which has approved the study protocol, the information sheets (patient and family versions), and the informed consent sheets (patient and familiar versions) on May 6th 2009 (Prot. N. 20406/CE, Date 14/05/2009) and by the Ethics Committee of each participating unit.

### Informed consent form and information sheet

Eligible participants are asked to participate only after receiving a detailed explanation of the nature, scope, and possible consequences of the trial. Participants receive an informed consent document including both information about the study and the consent form to sign. This document contains all the elements required by the Guidelines of Good Clinical Practice and any additional elements required by local regulations. The document is in a language understandable to the participants and specified who informs the participant. The person informing the participant is a psychiatrist or a psychologist. After reading the informed consent document, the patient, or his/her legal representative, gives consent in writing. The patient's consent is confirmed at the time of the consent by the personally dated signature of the participant and by the personally dated signature of the person conducting the informed consent discussion.

According to the Guideline of Good Clinical Practice, participants enrolled in the trial with the consent of the participants' legally acceptable representative are informed about the trial to the extent compatible with the participants' understanding and, if capable, the participant is asked to sign and personally date the written informed consent.

Participating patients are asked to give consent for the involvement of their family members in the study and those providing consent receive an informed consent document that includes both information about the study and the consent form that is given to family members. The staff member or the researcher informing family members is a psychiatrist or a psychologist. After reading the informed consent document, the participant gives consent in writing.

## Author contributions

MR conceived the project, coordinated all its phases and wrote the paper. AL collaborated in conceiving the project, coordinated the data collection, and had an important role in writing the paper. PS, MM, SS, STor, AF coordinated the data collection and revised the final draft. CB planned and realized the methodological protocol of the project, coordinated the realization of the on-line system for data collection, planned, and executed statistical analyses and contributed in writing the manuscript. DC realized the on-line system for data collection, merged data from all centers, checked data quality and prepared the dataset for statistical analyses. FP, EL, STos, KDS, CCo, STom, CCr, GP collaborated in data collection and revised the final draft.

## Funding

Ministry of Health, Italy—Ricerca Sanitaria Finalizzata, Code H61J08000200001.

### Conflict of interest statement

The authors declare that the research was conducted in the absence of any commercial or financial relationships that could be construed as a potential conflict of interest.
